# Supplementary material for: Divergent DNA methylation dynamics in marsupial and eutherian embryos
Source: Nature. 2025 May 14;642(8069):1073–9. doi: 10.1038/s41586-025-08992-2 (PMC12221971; doi:10.1038/s41586-025-08992-2)
Supplement: Supplementary file 2 — Reporting Summary [file 41586_2025_8992_MOESM2_ESM.pdf]

nature portfolio

Corresponding author(s): James MA Turner, Bryony J Leeke

Last updated by author(s): 20/1/25

Reporting Summary

Nature Portfolio wishes to improve the reproducibility of the work that we publish. This form provides structure for consistency and transparency in reporting. For further information on Nature Portfolio policies, see our Editorial Policies and the Editorial Policy Checklist.

Statistics

For all statistical analyses, confirm that the following items are present in the figure legend, table legend, main text, or Methods section.

n/a	Confirmed	
☐	☒	The exact sample size (*n*) for each experimental group/condition, given as a discrete number and unit of measurement
☒	☐	A statement on whether measurements were taken from distinct samples or whether the same sample was measured repeatedly
☐	☒	The statistical test(s) used AND whether they are one- or two-sided<br>*Only common tests should be described solely by name; describe more complex techniques in the Methods section.*
☒	☐	A description of all covariates tested
☒	☐	A description of any assumptions or corrections, such as tests of normality and adjustment for multiple comparisons
☐	☒	A full description of the statistical parameters including central tendency (e.g. means) or other basic estimates (e.g. regression coefficient) AND variation (e.g. standard deviation) or associated estimates of uncertainty (e.g. confidence intervals)
☐	☒	For null hypothesis testing, the test statistic (e.g. *F*, *t*, *r*) with confidence intervals, effect sizes, degrees of freedom and *P* value noted<br>*Give P values as exact values whenever suitable.*
☒	☐	For Bayesian analysis, information on the choice of priors and Markov chain Monte Carlo settings
☒	☐	For hierarchical and complex designs, identification of the appropriate level for tests and full reporting of outcomes
☒	☐	Estimates of effect sizes (e.g. Cohen's *d*, Pearson's *r*), indicating how they were calculated

Our web collection on statistics for biologists contains articles on many of the points above.

Software and code

Policy information about availability of computer code

Data collection	No software was used.
Data analysis	Code used in preparation and analysis of data and the generation of figures is available at github.com/bleeke.<br>Software used:<br>FastQC 0.11.5<br>TrimGalore 0.6.0<br>SAMTools 1.4<br>SNPsplit 0.3.4<br>BWA-MEM<br>BCFTools<br>Varscan<br>GATK<br>BEDtools<br>Fiji/ImageJ 2.0.0<br>Bismark 0.18.0<br>HISAT2 2.1.0<br>telescope 1.0.3<br>Nextflow/23.10.0<br>Nextflow/21.04.0<br>Singularity/3.6.4<br>Deeptools

April 2023

RNA-seq nf-core pipeline v3.2 and 3.12
methylseq nf-core pipeline 2.5.0
R 3.6.0 and 4.2.2
R packages
ggplot2 3.2.0
methylKit 1.4.1
genomicRanges 1.30.3
DESeq2 1.36
Rsubread 1.28.1
scater 1.14.6
Seurat 4.3.0

For manuscripts utilizing custom algorithms or software that are central to the research but not yet described in published literature, software must be made available to editors and reviewers. We strongly encourage code deposition in a community repository (e.g. GitHub). See the Nature Portfolio guidelines for submitting code & software for further information.

Data

Policy information about availability of data

All manuscripts must include a data availability statement. This statement should provide the following information, where applicable:

- Accession codes, unique identifiers, or web links for publicly available datasets
- A description of any restrictions on data availability
- For clinical datasets or third party data, please ensure that the statement adheres to our policy

BS-seq and RNA-seq data have been deposited at GE (accession number GSE206499). WGS data has been deposited at SRA (accession number PRJNA819000). Additional publicly available datasets used in this paper are accessible at GEO (GSE163620, GSE71434, GSE101571, GSE163620 and GSE71985), DDBJ (DRA006642 and DRA000570), and ArrayExpress (E-MTAB-7515). Reference genomes mm10, MonDom5 and ASM229v1 were accessed from Ensembl (https://www.ensembl.org/index.html) and mouse strain variants from the Mouse Genomes Project (https://www.sanger.ac.uk/data/mouse-genomes-project/)

Research involving human participants, their data, or biological material

Policy information about studies with human participants or human data. See also policy information about sex, gender (identity/presentation), and sexual orientation and race, ethnicity and racism.

Reporting on sex and gender	NA
Reporting on race, ethnicity, or other socially relevant groupings	NA
Population characteristics	NA
Recruitment	NA
Ethics oversight	NA

Note that full information on the approval of the study protocol must also be provided in the manuscript.

Field-specific reporting

Please select the one below that is the best fit for your research. If you are not sure, read the appropriate sections before making your selection.

☒ Life sciences ☐ Behavioural & social sciences ☐ Ecological, evolutionary & environmental sciences

For a reference copy of the document with all sections, see nature.com/documents/nr-reporting-summary-flat.pdf

Life sciences study design

All studies must disclose on these points even when the disclosure is negative.

Sample size	Sample sizes were not predetermined for opossum embryo BS-seq or scNMT-seq. The number of samples for each timepoint was dictated by litter size upon collection. Where sample availability permitted, for key timepoints we included multiple litters from separate collections. We ensured a minimum of 3 embryos of each sex at each timepoint. Our analysis of genomic coverage level at different genomic features supports our low input rare sample approach, as does our replication of previously reported mouse blastocyst methylation levels from similar low-input preparations. For opossum adult BS-seq, we collected 3 male and 3 female samples per tissue. For mouse adult BS-seq, we collected 3 male and 3 female samples per tissue. Mouse sperm and blastocyst BS-seq: sperm n=2 libraries of ~100 , E3.5 embryos n= 3.

Data exclusions	Some low-input BS-seq samples failed to amplify libraries and were therefore not further analysed. These are therefore not included in the final n number.
Replication	For opossum BS-seq we did not replicate over and above the initial number of collected samples due to limited availability of opossum embryo samples. Where sample availability permitted, for key timepoints we included multiple litters from separate collections and library preparations, and after QC in silico grouped all samples from the same timepoint for further analysis. We included mouse embryo, sperm, and brain BS-seq samples as a replication of prior published mouse BS-seq experiments as a validation of the method. For opossum scNMT-seq we collected > 200 single cells, resulting in >50 cells per timepoint/lineage, and analysed the single cells separately, showing the range of the data across all single cells.
Randomization	There were no experimental groups as this was a wild-type time-course study.
Blinding	There were no group allocations in this study (as explained in 'Randomization' above).

Reporting for specific materials, systems and methods

We require information from authors about some types of materials, experimental systems and methods used in many studies. Here, indicate whether each material, system or method listed is relevant to your study. If you are not sure if a list item applies to your research, read the appropriate section before selecting a response.

Materials & experimental systems

n/a	Involved in the study
☐	☒ Antibodies
☐	☒ Eukaryotic cell lines
☒	☐ Palaeontology and archaeology
☐	☒ Animals and other organisms
☒	☐ Clinical data
☒	☐ Dual use research of concern
☒	☐ Plants

Methods

n/a	Involved in the study
☒	☐ ChIP-seq
☒	☐ Flow cytometry
☒	☐ MRI-based neuroimaging

Antibodies

Antibodies used	#75-268, Anti 5hMeC, NeuroMab (1:1000)<br>#07-442, H3K9me3, MerckMillipore (1:200)<br>#ab1791, H3, Abcam, (1:100)<br>#BI-MECY-0100, 5MeC, Eurogentec (1:100)
Validation	We validated the use of the above antibodies by reproducing staining patterns previously published in mouse embryos (Extended Data Figure 2b), before applying them to opossum embryos.

Eukaryotic cell lines

Policy information about cell lines and Sex and Gender in Research

Cell line source(s)	Fibroblasts were derived from a male opossum neonate from our colony and immortalised by SV40-tag virus infection.
Authentication	Cell lines were not authenticated as they were derived by us.
Mycoplasma contamination	Cells were routinely tested for mycoplasma infection and found to be negative.
Commonly misidentified lines<br>(See ICLAC register)	No commonly misidentified cell lines were used in this study.

Animals and other research organisms

Policy information about studies involving animals; ARRIVE guidelines recommended for reporting animal research, and Sex and Gender in Research

Laboratory animals	Grey short-tailed opossum: Monodelphis domestica gametes, embryos from embryonic day 1.5- 7.5, adult (> 6 months < 2 years).<br>Mice: Mus musculus and Mus musculus x Mus spretus F1 cross. Embryos from embryonic day 3.5, adults (> 2 months < 6 months).
Wild animals	No wild animals were used.
Reporting on sex	For opossum embryos, we collected all embryos from each litter, but ensured a minimum of three embryos of each sex. Sex was

Reporting on sex	inferred based on ratio of reads mapping the X and pseudoY chromosome (Extended Data Figure 5g). For adult experiments, we collected 3 male and 3 female opossums and sex was identified based on physical sex characteristics.
Field-collected samples	No samples were collected from the field.
Ethics oversight	Opossums and mice were maintained in the Francis Crick Institute Biological Research Facility in accordance with United Kingdom Animal Scientific Procedures Act 1986 regulations (Project Licence P8ECF28D9) and subject to Francis Crick Institute ethical review. Additional opossums were housed at the University of Texas Rio Grande Valley under IACUC protocol AUP-19-31.

Note that full information on the approval of the study protocol must also be provided in the manuscript.

Plants

Seed stocks	NA
Novel plant genotypes	NA
Authentication	NA
